# Media representations of postpartum depression in UK newspapers (2020–2025): A thematic content analysis

**DOI:** 10.1007/s00127-026-03198-x

**Published:** 2026-09-08

**Authors:** Mustari Sarkar Trisha, Mahmudul Hasan Nahid, Rachael L Murray

**Affiliations:** 1 School of Medicine, University of Nottingham, Nottingham, UK; 2 University Hospitals Leicester (Acute Medicine), Leicester, UK; 3 School of Medicine, University of Nottingham, Nottingham, UK

**Keywords:** Postpartum depression, Media representation, Newspaper coverage, Maternal mental health, Thematic content analysis, PPD

## Abstract

**Purpose:**

Postpartum depression (PPD) is a common maternal mental health disorder, affecting up to one in six mothers, with serious consequences for maternal well-being, child development, and economic costs. Media shape public mental health perceptions, and newspapers remain an important information source. Yet systematic analyses of UK newspaper coverage of PPD are scarce. This study examined representations in national newspapers (2020–2025), comparing broadsheets and tabloids to identify themes and shifts over time.

**Methods:**

A qualitative content analysis was conducted using LexisNexis (a commercial database that aggregates full-text news content from national and international print and online media) to retrieve six widely read and influential national newspapers (*The Guardian*,* The Independent*,* The Times*,* Daily Mirror*,* The Sun*,* Daily Mail*) from 2020 to 2025. Guided by a social constructionist framework, articles meeting inclusion criteria were analysed at a latent level using inductive reflexive thematic analysis following Braun and Clarke’s six-phase approach.

**Results:**

From 1,425 records, 68 articles met the inclusion criteria and five themes were identified: shifting awareness of postpartum depression, stigma and silence around disclosure, maternal mental health services and support, the impact of COVID-19, and lived experiences of distress, recovery and cultural representation. Coverage overall appeared to move from early minimisation toward greater recognition, with reporting highest in 2023 before declining in subsequent years; this pattern should be interpreted cautiously given the short timeframe and modest sample. Broadsheets provided most reporting, often highlighting systemic failings and inequalities, while tabloids contributed fewer articles, relying largely on emotive personal stories and celebrity accounts.

**Conclusion:**

UK newspaper portrayals increasingly acknowledged postpartum depression but continued to perpetuate stigma and expose weaknesses in support systems. The findings illustrate the media’s dual influence in validating maternal mental health while sustaining obstacles, offering implications for awareness, stigma reduction, and policy development.

**Supplementary Information:**

The online version contains supplementary material available at https://doi.org/10.1007/s00127-026-03198-x.

## Introduction

Postpartum depression (PPD) is a perinatal mood disorder and one of the most common complications of childbirth, affecting approximately 17.22% of mothers worldwide [1]. In this paper, the widely used clinical and lay term “postpartum depression” is retained throughout, consistent with prevailing NHS and Royal College of Psychiatrists usage of “postnatal depression” for the same condition, while recognising that diagnostic classification situates it within the broader category of perinatal depression, as set out below. The *Diagnostic and Statistical Manual of Mental Disorders* (DSM-5) classifies PPD within perinatal depression, defining it as a major depressive episode arising during pregnancy or within four weeks postpartum [2]. Many scholars, however, advocate extending the diagnostic window to 12 months to capture delayed onset [3]. Symptoms typically include persistent low mood, irritability, anhedonia, anxiety, sleep disturbance, feelings of worthlessness, and impaired maternal–infant bonding; suicidal ideation may occur in severe presentations [4, 5]. These features can substantially undermine daily functioning and the capacity to care for an infant.

The burden of PPD is marked both globally and in the UK. In England, recent estimates suggest that 56,000–85,000 new mothers (10–15% of those giving birth) were affected in 2024 [6, 7]. Meta-analytic evidence indicates a pooled prevalence near 17% among women without prior depression [8], with other syntheses reporting rates in the 13–19% range across settings and methodologies [8, 9]. PPD is now understood as multifactorial, driven by biological, psychological, and social determinants. Established risks include socioeconomic adversity, limited social support, obstetric complications, and personal or family psychiatric history [10]. Evidence from umbrella reviews further identifies exposure to violence, gestational diabetes, caesarean section, postpartum sleep disruption, and vitamin D deficiency as salient risks, while protective factors include early skin-to-skin contact and adequate maternal nutrition [11]. These patterns underline the need for prevention strategies that operate across clinical, social, and behavioural domains.

Consequences extend well beyond the immediate postpartum period. Affected mothers frequently report reduced self-efficacy, social withdrawal, and diminished health-related quality of life, with qualitative accounts evoking the sense of being “trapped in a dark hole,” capturing the depth of distress [12, 13]. PPD is also associated with difficulties in early interactions and caregiving routines [14]. For children, exposure to maternal depression has been associated with disrupted attachment relationships and developmental risks across language, cognitive, and socio-behavioural domains, with evidence also linking persistent maternal depression to emotional dysregulation and longer-term disadvantages such as poorer academic outcomes [15–18]. These intergenerational effects emphasise the public health significance of timely identification and support.

Economic and societal costs are substantial. UK analyses estimate perinatal mental health problems cost approximately £8.1 billion annually, over 70% of which reflects adverse outcomes for children and associated service use in health, education, and social care [19]. Lifetime costs per affected woman have been estimated to exceed £70,000 [20]. Beyond economics, maternal suicide remains a leading cause of perinatal mortality in high-income countries, indicating critical gaps in detection and crisis response [21]. Although effective treatments, including psychological therapies and antidepressant medication, are available, uptake remains limited, with a number of barriers to help-seeking and access to care identified in the literature [22–23]. The media shape how the public understands mental health, and newspapers have traditionally played an influential role in agenda setting and health information dissemination [24, 25], although it is acknowledged that this influence is evolving as print circulation declines and digital and social media grow. Representations of PPD can either promote empathy and early help-seeking or reinforce stigma and misinformation [24–26]. Evidence indicates that sensationalised or inaccurate portrayals deter care-seeking, whereas supportive coverage improves attitudes and mental health literacy [26]. Given that PPD is already susceptible to delays in diagnosis due to cultural expectations, limited awareness, and self-stigma, public-facing communication through trusted media channels becomes central to stigma reduction and awareness-raising [24, 27].

Despite this influence, PPD-specific analyses of UK newspaper coverage remain limited. Much of the existing research considers mental illness broadly rather than PPD as a distinct condition [25, 28]. Few studies examine PPD directly, and fewer analyse how newspaper types frame it. Previous research examining UK newspaper coverage of mental health has found that reporting often focuses on individualised narratives and dramatic events while giving limited attention to structural issues such as healthcare provision or policy context. For example, a longitudinal content analysis of nine UK national newspapers published between 2008 and 2019 identified persistent differences in tone and framing across tabloid and broadsheet reporting of mental illness [29]. In addition, media scholarship highlights how motherhood is frequently framed through simplified moral binaries that position mothers as either resilient and devoted or negligent and failing, limiting more nuanced representations of maternal mental health experiences [30]. Moreover, media ecosystems are heterogeneous: broadsheets and tabloids differ in editorial priorities, rhetorical style, and moral framing, likely producing divergent public narratives [31]. These divergent narratives matter because they may carry different implications for public stigma and help-seeking around maternal mental health. Yet systematic UK comparisons of broadsheet versus tabloid portrayals of PPD are scarce, leaving open questions about how editorial logics shape maternal mental health narratives and, ultimately, how media environments may facilitate or impede early identification and stigma reduction.

Against this backdrop, this study aims to analyse how postpartum depression (PPD) is represented in UK newspaper coverage between 2020 and 2025 by identifying dominant themes and exploring the narratives surrounding maternal mental health, comparing representations across broadsheet and tabloid newspapers, and examining shifts in coverage over time. By doing so, the research contributes to improving public health messaging and may inform the design of media-sensitive interventions, campaigns, and maternal mental health policies.

## Methodology

This study was grounded in a social constructionist framework, which assumes that realities are produced through social interaction, cultural norms, and language, rather than existing as objective facts [32]. In the case of PPD, this approach recognises that the condition is not solely a clinical diagnosis but also a socially mediated phenomenon, shaped by cultural expectations of motherhood, stigma surrounding maternal mental illness, and dominant media narratives [32, 33]. The dataset was generated through a systematic search of UK national newspapers using LexisNexis©, undertaken on 1st July 2025. The timeframe was set from 1st January 2020 to 30th June 2025 to allow the capture of trends over time, including COVID-19. Search terms included “postpartum depression,” “postnatal depression,” “perinatal depression,” “PPD,” “maternal depression,” and “baby blues.” Six newspapers were purposively selected: three tabloids (The Sun, Daily Mirror, Daily Mail) and three broadsheets (The Guardian, The Independent, The Times). These titles were chosen because they are among the most widely read and influential in the UK, with the Daily Mail and The Sun maintaining circulations of over 600,000 as of mid-2025 [34]. Articles were retrieved as they appear in LexisNexis’s aggregated archive, which primarily draws on print and online-syndicated content, rather than newspapers’ digital-only or web-exclusive articles.

Articles were included according to the criteria described in Table 1. No formal quality appraisal was applied, as the included articles constitute the data under study rather than evidence sources to which appraisal tools would apply. Duplicates and near-identical versions were addressed through a three-step process: the version with higher readership metrics was prioritised; if readership was identical, the earliest publication date was selected; and if both were identical, one was chosen randomly using random.org.

**Table 1:** Inclusion and exclusion criteria applied during article screening

Criterion type	Criteria
Inclusion	Newspaper articles provided a substantive discussion of PPD as a central focus
Inclusion	Published in English, in one of the six selected UK national titles, 1st Jan 2020–30th Jun 2025
Exclusion (a)	Only passing reference to PPD without substantive discussion
Exclusion (b)	Discussed related conditions (e.g. general depression, perinatal anxiety) without focusing on PPD
Exclusion (c)	Opinion pieces, letters, or short snippets under 300 words

Articles retrieved from LexisNexis were exported into Microsoft Word for initial familiarisation. Metadata (publication title, date, source, word count) were systematically logged in an Excel database, which also supported inclusion and exclusion tracking, duplicate management, and classification by newspaper type (tabloid/broadsheet) and publication year. Excel additionally functioned as an audit trail by linking coding decisions and emerging themes with article metadata. Final coding was managed in Word, with relevant extracts collated into theme-specific files for detailed analysis.

Thematic analysis was undertaken in line with Braun and Clarke’s six-phase framework [35], using a latent, inductive approach. Given the limited prior research on PPD in UK media, inductive coding was considered appropriate to ensure that patterns were grounded in the data rather than predefined categories. The analytic process comprised repeated reading, manual coding, collation of codes into provisional themes, iterative refinement, and integration into an interpretive narrative. Themes were reviewed by another researcher [RM] to enhance credibility and reduce potential bias. Particular attention was directed to differences between tabloid and broadsheet coverage and to changes across the 2020–2025 period.

Reporting adheres to ENTREQ and SRQR standards [36, 37]. Representative article extracts are included to illustrate key interpretive claims. The University of Nottingham Research Ethics Committee granted ethical approval for the study.

## Results

### Characteristics of included articles

**Fig. 1:**
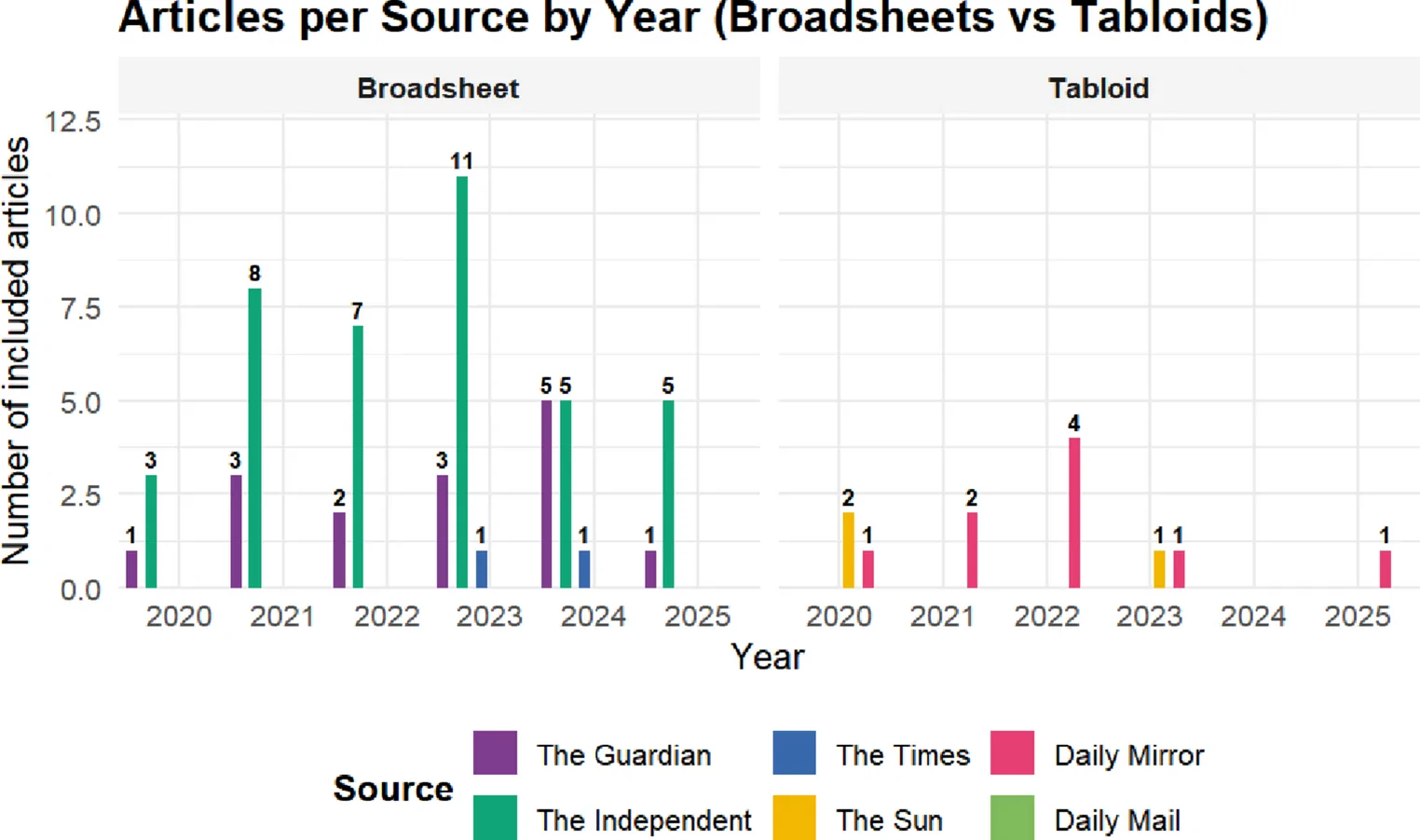
Number of articles included in the study each year by source

A total of 1,425 articles were identified in the initial search. After applying inclusion and exclusion criteria, 68 were retained for analysis. Coverage was highest in 2023 before declining in later years. National broadsheets provided most articles, particularly *The Independent* and *The Guardian*, with fewer contributions from *The Times*. Within the tabloids, most articles were drawn from the *Daily Mirror*, with limited representation from *The Sun*. Although *Daily Mail* articles were retrieved, none satisfied the inclusion criteria. Figure 1 summarises the distribution of included articles by year and newspaper type; Table 2 the relative emphasis of themes across broadsheet and tabloid newspapers.

**Table 2:** Relative emphasis of each theme across broadsheet and tabloid coverage

Theme	Broadsheet emphasis	Tabloid emphasis	Pattern of coverage
1. Shifting awareness	Strong	Limited	Broadsheets amplified political voices, campaigns and paternal testimony; tabloid awareness emerged mainly through celebrity accounts
2. Stigma and silence	Moderate	Strong	Broadsheets emphasised racial/cultural inequalities in stigma; tabloid coverage more directly linked disclosure to shame and fear of child removal
3. MMH services and support	Strong	Moderate	Broadsheets focused on underfunding and staff shortages; tabloids emphasised long waits and risk to mothers
4. PPD in the shadow of COVID-19	Strong	Limited	Isolation, exhaustion, and economic-cost framing were drawn predominantly from broadsheet reporting
5. Living with PPD	Strong	Moderate	Broadsheets carried most cultural/critical portrayals (film and theatre reviews); tabloids contributed celebrity and ordinary-mother testimony

Note: Ratings (strong/moderate/limited) are qualitative characterisations of the relative prominence of each theme within broadsheet and tabloid coverage, based on the thematic analysis rather than article counts

### Thematic content analysis

Five themes and thirteen corresponding subthemes were identified and grouped into two categories: those addressing how PPD was represented in newspapers, and those highlighting the structural and systemic contexts shaping maternal mental health.

**Fig. 2:**
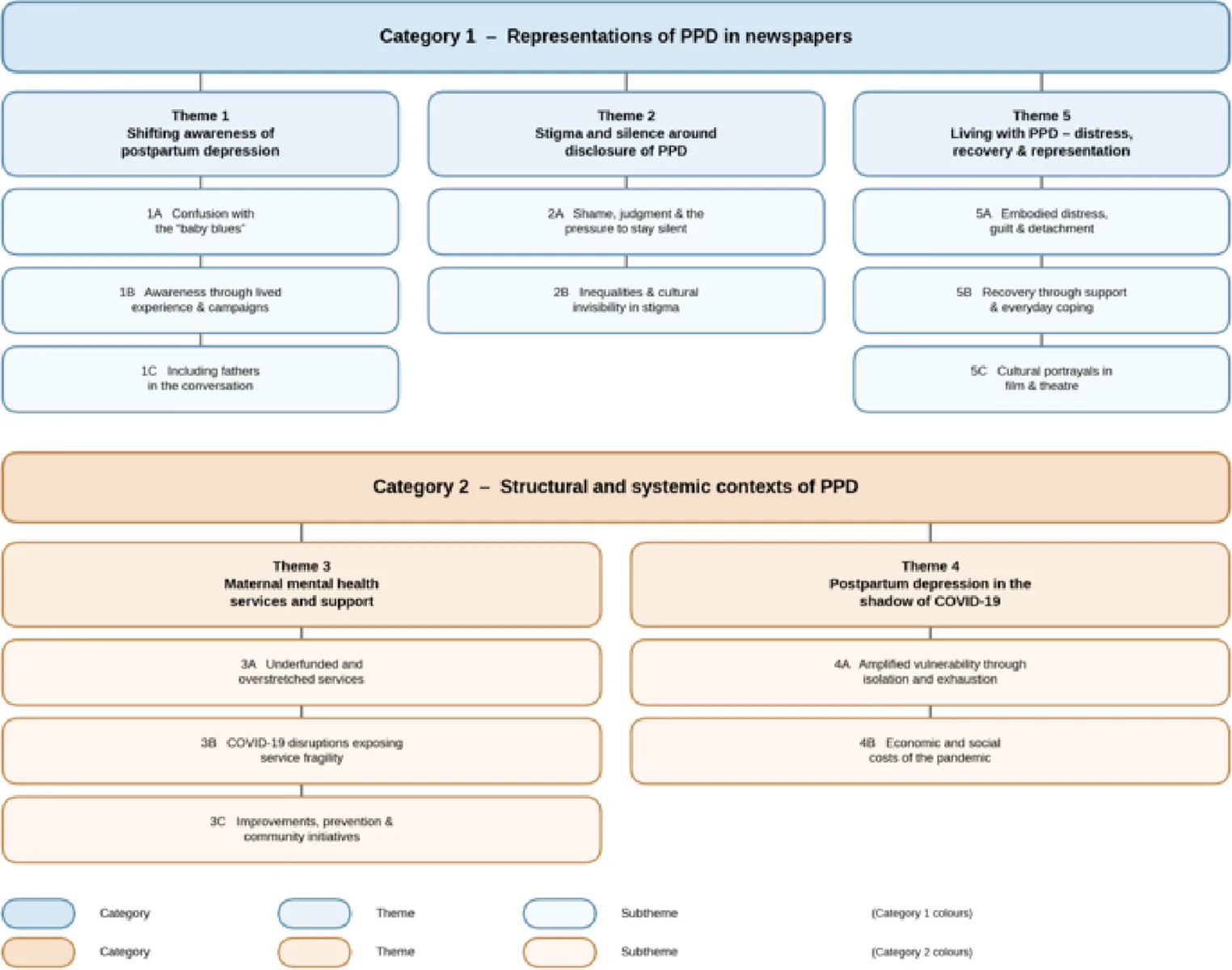
Thematic Map of UK Newspaper Coverage of Postpartum Depression (PPD) (2020–2025) *Category,* theme*,* and subtheme boxes are distinguished by size and by an outer border colour (blue for Category 1*,* amber for Category 2)*,* with pale fill and black text throughout for readability.* PPD = postpartum depression; COVID-19 = coronavirus disease 2019

A visual map of the themes and corresponding subthemes is provided in Fig. 2.

### Theme 1: Shifting awareness of postpartum depression

#### 1 A: Confusion with the “baby blues”

Early coverage often blurred the boundary between short-term mood changes and clinical depression, which minimises the seriousness of PPD. Some reports portrayed it as ordinary sadness, while others sought to clarify the distinction by listing symptoms such as persistent sadness, anxiety, and exhaustion. As *The Independent* (24th June 2023) explained, “Most new mothers experience what is known as the ‘baby blues’… but postnatal depression is both more severe and longer lasting.”, illustrating how newspapers attempted to correct misunderstandings while acknowledging ongoing public confusion.

#### 1B: Awareness through lived experience and campaigns

By the mid-2020s, newspaper reporting increasingly presented PPD as under-recognised but serious in its consequences for mothers and families. Broadsheets frequently amplified political voices and public campaigns, portraying the condition as a pressing health concern requiring greater visibility and earlier identification. Reports drew on both lived experience and expert perspectives to legitimise PPD, whereas tabloids engaged less, with awareness emerging primarily through celebrity accounts.

“I was astonished at how little is understood about the condition.” (Kate Forbes, *The Independent*, 24^th^ June 2024) “It’s good they are bringing up postnatal depression. It’s a very real thing, and so many women endure it in silence.” (*The Independent*, 5^th^ December 2024).

#### 1 C: Including fathers in the conversation

Later coverage broadened recognition of PPD to include fathers as well as mothers. Broadsheets highlighted paternal testimonies, emphasising that support systems often overlooked men’s experiences. Tabloid reporting contributed less, though references to men’s peer-support groups such as Andy’s Man Club suggested growing acknowledgment of paternal struggles.

“Both parents can experience postnatal depression.” (*The Independent*, 20^th^ January 2025) “Men are not expected to be interested in babies… what might have been different if he had been viewed as someone who needed help too?… I don’t think I would have ended up in hospital.” (*The Guardian*, 31^st^ May 2025) “I’d heard about a local men’s mental health group called Andy’s Man Club, … It was nerve-wracking, but when I heard other men talk about experiencing similar feelings I realised I wasn’t alone” (*The Sun*, 9^th^ August 2020).

### Theme 2: Stigma and silence around disclosure of PPD

#### 2 A: Shame, judgment, and the pressure to stay silent

Newspapers frequently depicted stigma as a reason women remained silent about PPD. Coverage showed how embarrassment positioned PPD as a personal weakness rather than a health condition, with both ordinary mothers and celebrities emphasising pressure to appear as though they were coping. Alongside shame, fear of judgment created further barriers, with reporting suggesting disclosure could risk losing parental responsibility. This was reinforced in tabloid coverage, where reluctance to seek medical help was linked to anxiety about child removal.

“Suffering with depression was the hardest because you have to then own up to what you’re going through.” “For ages, it felt embarrassing…Talking about it makes you look crazy.” (The Independent, 9^th^ August 2022).

“I don’t know why women have to feel as if they have got all their s*** together… I felt ashamed of myself feeling that way.” (Georga Kousoulou, The Independent, 8^th^ September 2022).

“Afraid… her baby would be taken away if she disclosed her depression.” (The Independent, 24^th^ June 2023).

“Reluctant to go to the doctors because they’re always afraid they’ll take the kids away.” (Daily Mirror, 2021).

#### 2B: Inequalities and cultural invisibility in stigma

Some coverage highlighted how stigma was unevenly experienced, with broadsheets drawing attention to racial and cultural inequalities. Reports suggested Black mothers were often dismissed or denied adequate emotional support, showing how stigma could be reinforced by systemic neglect, with *The Guardian* (23rd October 2024) noting that “Black mothers are often not taken seriously, nor offered emotional support.” Such reporting stressed that stigma was not evenly distributed, with racial and cultural marginalisation shaping how support was offered or withheld. 

### Theme 3: Maternal mental health services and support.

#### 3 A: Underfunded and overstretched services

Newspapers frequently described perinatal mental health care as insufficient, framing shortages as a structural problem rather than an individual failing. Reports highlighted how provision varied across regions, creating barriers to timely access. Broadsheets often drew attention to underfunding and staff shortages, while tabloids emphasised long waits and the risks posed to new mothers. Together, these accounts situated maternal distress within the wider context of service neglect.

“Perinatal care [is] dangerously patchy and unreliable. Access… remains a postcode lottery.” (The Guardian, 5^th^ December 2023).

“Almost 20,000 women a year… wait up to 19 months to start treatment.” (The Guardian, 5^th^ December 2023).

“Half of anxiety and depression cases among new and expectant mothers were being missed amid NHS staff shortages.” (The Independent, 11^th^ January 2024).

#### 3B: COVID-19 disruptions exposing service fragility

The pandemic exposed the vulnerability of maternal mental health care, with existing gaps made worse by widespread disruption. Mothers described how support “fell away immediately, replaced with short phone calls where I felt too shellshocked… to even open my mouth” (*The Independent*, 6th April 2021). Specialist services were also withdrawn, with reports noting that midwifery provision in some regions “stopped” altogether (*The Independent*, 28th July 2021). These accounts highlighted how fragile maternal mental health services were, collapsing at the very moment when need intensified.

#### 3 C: Improvements, prevention, and community initiatives

Alongside critique, newspapers also drew attention to incremental improvements and new forms of support. Pharmaceutical advances were presented as breakthroughs, including the FDA approval of a pill “specifically intended to treat severe depression after childbirth,” supported by research showing the effectiveness of SSRIs in reducing both maternal symptoms and child difficulties (*The Independent*, 4th August 2023).

Preventive strategies also featured in coverage, with exercise described as lowering risk by 45% (*The Guardian*, 7th November 2024) and the newly introduced routine “physical and mental health check six weeks after giving birth”, reflecting that year’s introduction of a dedicated maternal postnatal consultation to the GP contract (*The Guardian*, 7th February 2020).

Innovation beyond statutory care was highlighted through digital and grassroots responses. Initiatives ranged from an “AI chatbot… to support early detection” (*The Times*, 15th January 2024) to community projects like Mama Moments, which promoted self-care as protection for mothers’ mental health (*Daily Mirror*, 26th November 2020). These examples suggested that while NHS provision remained inconsistent, alternative supports were increasingly valued as part of the maternal mental health landscape.

Theme 4: Postpartum depression in the shadow of COVID-19.

#### 4 A: Amplified vulnerability through isolation and exhaustion

Reporting consistently framed the pandemic as an intensifying risk for PPD. Evidence pieces cited studies showing that “new mothers were twice as likely to suffer from postnatal depression in lockdown,” with “low social support” and barriers to practical help leaving many “totally overwhelmed” (*The Independent*, 11th May 2021). First-person testimonies echoed these mechanisms: one mother recalled, “emotionally, I fell apart… I felt trapped, unfulfilled, wistful and utterly alone” (*The Independent*, 22nd March 2021).

#### 4B: Economic and social costs of the pandemic

Broadsheet coverage also linked maternal mental health during COVID-19 to wider social and economic consequences. Disruption to maternity services was framed not only as a personal burden but as a collective issue, with estimates suggesting it could “cost an extra £17.5bn in England” (*The Independent*, 28th July 2021). By presenting PPD in economic as well as health terms, newspapers positioned maternal wellbeing as a matter of national importance, reinforcing its relevance for public health and policy agendas.

Theme 5: Living with PPD – distress, recovery, and representation.

#### 5 A: Embodied distress, guilt, and detachment

Accounts of PPD emphasised its profound emotional and relational toll. Newspapers highlighted feelings of exhaustion, detachment, and guilt, presenting PPD as something that affected both the body and relationships with infants. Celebrities described how depression disrupted daily life, while ordinary mothers echoed these struggles, emphasising difficulties bonding and the shame that followed. Together, these perspectives conveyed how PPD was portrayed:

“Debilitating… I wanted the world to stop.” (Jess Wright, The Independent, 9^th^ May 2025).

“I have experienced it. Twice… the first time was very difficult, the second more manageable.” (Kylie Jenner, The Independent, 22^nd^ February 2023).

“I didn’t really feel the love for him… for at least a year to 18 months.” (The Independent, 2^nd^ May 2025).

#### 5B: Recovery through professional support and everyday coping

Recovery was portrayed as a varied, ongoing process, combining professional help with everyday strategies that offered comfort and resilience. Formal treatments such as therapy and medication were described as vital first steps for many, though often accessed only after struggling in silence. The difficulty of seeking such help was also emphasised, with medication depicted as a heavy burden rather than a simple act. Creative and preventive activities were likewise presented as important tools for regaining selfhood and protecting mental health.

“After 18 months of struggling in silence, I reached out… therapy was the best thing I’ve ever done.” (Daily Mirror, 17^th^ June 2025).

“CBT gave me coping strategies… psychotherapy… helped me get to the root.” (Daily Mirror, 24^th^ October 2023).

“Sometimes reaching for your medication is like picking up a 60kg dumbbell.” (Chrissy Teigen, Independent, 5^th^ March 2020).

“Pottery gave me a whoosh of positivity… for that moment, I felt like myself again… it felt like therapy.” (Florence St George, The Independent, 21^st^ November 2021).

#### 5 C: Cultural portrayals in film and theatre

Cultural media also shaped how PPD was understood, with theatre and film presenting it in ways that both raised awareness and risked sensationalism. Theatre reviews highlighted stark depictions of maternal ambivalence, such as one play that included the line, *“I didn’t know I could both love and hate something so fully… I get why people shake their babies”* (*The Independent*, 27th October 2021). Film reviews of *A Mouthful of Air* similarly portrayed maternal suffering, describing it as “a painful, intimate, sincerely intended study,” while also warning that it was “walking on eggshells” (*The Guardian*, 8th February 2022). These representations reinforced the cultural visibility of PPD but left questions about whether dramatic portrayals sensitively conveyed women’s experiences or risked reducing them to dramatic effect.

## Discussion

### Summary of findings and interpretation

This study examined how PPD was represented in UK newspapers between 2020 and 2025, focusing on dominant narratives, differences between broadsheets and tabloids, and shifts over time. Five broad themes were identified: awareness, stigma, systemic shortcomings, the impact of COVID-19, and portrayals of distress and recovery.

Awareness of PPD was uneven. Earlier reports often blurred it with the “baby blues,” downplaying severity, while later coverage drew on lived experiences, campaigns, and paternal voices to present it as a serious condition. Broadsheets, particularly *The Independent* and *The Guardian*, carried most of this reporting, using it to highlight wider problems of health inequality and policy neglect. Tabloids, however, fewer in number, took a different approach, relying on emotional testimony and celebrity disclosure. This helped humanise PPD but also risked reducing it to individual struggles rather than systemic concerns.

Stigma was a persistent thread, framed in tabloids as shame and fear of child removal, while broadsheets exposed how it intersected with cultural and racial inequalities. Systemic failings, obvious during the pandemic, were portrayed as the result of fragile and underfunded services rather than individual weakness. Finally, recovery stories and cultural portrayals gave PPD visibility and helped normalise discussion, though dramatic framings sometimes tipped into sensationalism.

### Comparison with existing literature

Early conflation of postpartum depression (PPD) with the “baby blues” (Subtheme 1 A) reflects earlier analyses showing journalism often compresses diagnostic categories or defaults to shorthand labels [25, 28]. Yet while DSM-5-TR defines PPD as a major depressive episode with functional impairment, this distinction was rarely conveyed in reporting, minimising severity despite meta-analyses confirming its prevalence and impact [1, 3, 8, 9]. Unlike transient mood lability, untreated PPD is longer and more recurrent [8, 10]. Later coverage that highlighted persistence, disruption, and caregiving difficulties corresponded more directly with clinical evidence, extending research validating the Edinburgh Postnatal Depression Scale (EPDS) when embedded in diagnostic pathways [37, 38]. Attention also turned to fathers (Subtheme 1 C), echoing evidence that paternal depression affects 8–9% of men [39]. However, whereas clinical studies emphasise family-wide consequences, newspapers portrayed paternal experiences mainly as isolated testimonies, overlooking evidence that persistent parental depression increases socio-emotional and cognitive risks for children and underlining the need for family-inclusive prevention [40].

Themes highlighting stigma, shame, and fears of judgement (2 A-B), consistent with qualitative reviews showing internalised stigma discourages disclosure of depressive symptoms [23, 41]. Beck’s description of women “trapped” by motherhood ideals resonates with accounts of silence in reporting [5, 12]. Broadsheets’ emphasis on racialised invisibility (Subtheme 2B) reflected evidence that ethnic-minority women face poorer recognition and emotional support in perinatal care [42].

Systemic shortcomings (Theme 3 A), described as postcode lotteries and long waits, mirrored evidence of under-investment and societal cost [19, 20]. Although delayed treatment is known to prolong episodes and escalate crises, newspapers seldom connected these delays to PPD’s role in maternal mortality [21]. During COVID-19 (Themes 3B, 4), isolation and disrupted care echoed meta-analyses showing worsened maternal mood [43]. Broadsheets added a distinctive frame, emphasising economic loss (£17.5 bn), extending cost estimates by linking financial burden directly to pandemic disruption [20].

Where newspapers proposed solutions (Theme 3 C), this broadly mirrored evidence. Psychological interventions (CBT, IPT) are effective and stepped-care models such as TRIPPD improve engagement [44]. Peer and health-visitor programmes reduce risk, while online interventions like MumMoodBooster show symptom reduction [45, 46]. Preventive strategies, such as exercise, highlighted in reporting, are supported by reviews [47]. For moderate–severe cases, pharmacotherapy remains vital, with neuroactive-steroid agents Brexanolone and Zuranolone showing rapid efficacy [48]. Lived-experience accounts of guilt, detachment, and bonding difficulties (Theme 5) echoed qualitative studies, while recovery narratives reinforced evidence that community-based, multi-component interventions provide sustained benefit [12, 14, 44].

### Policy and practice implications

These findings have important implications for maternal mental health policy and practice. Persistent public uncertainty highlights the need for clearer public-facing messaging. Stigma, repeatedly reinforced in media narratives, must also be addressed structurally. Policy should embed anti-stigma education into perinatal care and ensure women can disclose without fear of judgment or child removal. Interventions combining lived experience with practical information have reduced internalised stigma and improved help-seeking [41].

Digital innovations, including AI chatbots and peer support, show promise; technology-enabled CBT and mobile platforms reduce symptoms and increase access, especially where services are limited [49]. However, these are most effective when integrated within stepped-care systems [44]. Finally, systemic shortcomings demand reform: underfunding, staff shortages, and postcode disparities heighten risk, whereas integrated, family-centred models improve outcomes. The media themselves also have a role to play: clinicians, professional bodies (e.g. the Royal College of Psychiatrists, Royal College of Obstetricians and Gynaecologists), and policymakers could work more directly with journalists and editors – through guidance on non-stigmatising language, structured clinician comment in reporting, and closer engagement with awareness campaigns – so that newspaper coverage supports earlier help-seeking rather than reinforcing stigma.

### Strengths and limitations

This study has several strengths. It is the first to systematically compare broadsheet and tabloid portrayals of postpartum depression (PPD) in the UK, offering new insight into how editorial styles shape public narratives. The dataset spans 2020–2025, capturing the COVID-19 pandemic, which heightened maternal vulnerability and public debate. Rigorous thematic analysis, supported by reflexivity, ensured transparency and minimised bias. By incorporating the voices of political leaders, clinicians, celebrities, and mothers, the analysis reflects diverse perspectives in national discourse.

Limitations must also be noted. Reliance on LexisNexis may have excluded digital-only outlets, introducing selection bias. Relatedly, in the absence of any appraisal framework applicable to journalistic texts, all included articles contributed equally to the analysis regardless of length or depth of reporting. The focus on six national titles excluded local and ethnic press, limiting transferability. As only published texts were analysed, audience reception was not assessed. Thematic interpretation remains shaped by researcher subjectivity, and the lack of triangulation with policy or clinical data restricts contextual depth.

### Future research

Future studies should extend beyond print to examine how digital platforms (e.g., Instagram, TikTok, parenting forums) construct postpartum depression, as these increasingly shape health information and informal peer support. Research should also assess audience reception, exploring how narratives shape perceptions, stigma, and help-seeking. Cross-national comparisons could test whether UK coverage mirrors other systems. Longitudinal designs may track whether shifts improve literacy or service uptake. Finally, intervention studies should evaluate AI chatbots, campaigns, and media guidelines in shaping maternal mental health outcomes.

## Conclusion

This study examined how UK newspapers represented postpartum depression (PPD) between 2020 and 2025, with attention to broadsheet–tabloid differences and shifts over time. Findings showed a move from minimisation and conflation with the “baby blues” towards greater recognition, though stigma and systemic shortcomings persisted. Broadsheets framed PPD through expert critique and health inequalities, while tabloids emphasised emotive testimonies and celebrity accounts. These complementary styles broadened visibility but sometimes reinforced barriers. The study highlights the media’s dual role in legitimising maternal mental health while sustaining obstacles, informing communication, stigma reduction, and policy reform.

## Supplementary Information

Below is the link to the electronic supplementary material.

Supplementary Material 1

## Data Availability

The datasets generated and analysed during the current study are derived from publicly available newspaper sources accessed via LexisNexis using University of Nottingham user access. Further details are available from the author upon reasonable request.
