# Supplementary material for: Media representations of postpartum depression in UK newspapers (2020–2025): A thematic content analysis
Source: Soc Psychiatry Psychiatr Epidemiol. 2026 Sep 8;61(12):2647–57. doi: 10.1007/s00127-026-03198-x (PMC13727838; doi:10.1007/s00127-026-03198-x)
Supplement: Supplementary file 1 [file 127_2026_3198_MOESM1_ESM.docx]

Appendix:

Study metadata:

Figure S1: Metadata of the included articles, including title, source, year & word count

Study selection process:

Figure S2: Flowchart of the study selection process, including their reasons
